# Addition of a dairy fraction rich in milk fat globule membrane to a high-saturated fat meal reduces the postprandial insulinaemic and inflammatory response in overweight and obese adults

**DOI:** 10.1017/jns.2015.42

**Published:** 2016-03-07

**Authors:** Elieke Demmer, Marta D. Van Loan, Nancy Rivera, Tara S. Rogers, Erik R. Gertz, J. Bruce German, Jennifer T. Smilowitz, Angela M. Zivkovic

**Affiliations:** 1 Department of Nutrition, University of California Davis, Davis, CA, USA; 2 USDA/ARS Western Human Nutrition Research Center, Davis, CA, USA; 3 Foods for Health Institute, University of California, Davis, CA, USA; 4 Department of Food Science & Technology, University of California, Davis, CA, USA

**Keywords:** Milk fat globule membrane, Postprandial inflammation, Saturated fat, Insulin, C-reactive protein, CVD, Cytokines, CRP, C-reactive protein, iAUC, incremental AUC, MetS, metabolic syndrome, MFGM, milk fat globule membrane, PO, palm oil, sICAM, soluble intracellular adhesion molecule

## Abstract

Meals high in SFA, particularly palmitate, are associated with postprandial inflammation and insulin resistance. Milk fat globule membrane (MFGM) has anti-inflammatory properties that may attenuate the negative effects of SFA-rich meals. Our objective was to examine the postprandial metabolic and inflammatory response to a high-fat meal composed of palm oil (PO) compared with PO with an added dairy fraction rich in MFGM (PO+MFGM) in overweight and obese men and women (*n* 36) in a randomised, double-blinded, cross-over trial. Participants consumed two isoenergetic high-fat meals composed of a smoothie enriched with PO with *v*. without a cream-derived complex milk lipid fraction ( dairy fraction rich in MFGM) separated by a washout of 1–2 weeks. Serum cytokines, adhesion molecules, cortisol and markers of inflammation were measured at fasting, and at 1, 3 and 6 h postprandially. Glucose, insulin and lipid profiles were analysed in plasma. Consumption of the PO + MFGM *v*. PO meal resulted in lower total cholesterol (*P* = 0·021), LDL-cholesterol (*P* = 0·046), soluble intracellular adhesion molecule (*P* = 0·005) and insulin (*P* = 0·005) incremental AUC, and increased IL-10 (*P* = 0·013). Individuals with high baseline C-reactive protein (CRP) concentrations (≥3 mg/l, *n* 17) had higher (*P* = 0·030) insulin at 1 h after the PO meal than individuals with CRP concentrations <3 mg/l (*n* 19). The addition of MFGM attenuated this difference between CRP groups. The addition of a dairy fraction rich in MFGM attenuated the negative effects of a high-SFA meal by reducing postprandial cholesterol, inflammatory markers and insulin response in overweight and obese individuals, particularly in those with elevated CRP.

The postprandial state has been highlighted as an important transitory period when significant vascular damage can occur and has recently been implicated in the causal processes of CVD^(^^1^^)^. Postprandial inflammatory and lipaemic responses are pronounced in individuals with obesity, the metabolic syndrome (MetS) and type 2 diabetes^(^^2^^–^^5^^)^, in part because the magnitude of the postprandial inflammatory response is correlated with insulin resistance^(^^6^^,^^7^^)^. Current literature suggests that the inflammation of the postprandial state adds to the already pro-inflammatory environment in individuals with obesity-induced metabolic disease, intensifying the overall systemic inflammation that underlies metabolic dysfunction^(^^8^^)^. Given the rise in overweight and obesity worldwide^(^^9^^)^, potential nutritional interventions that can limit the postprandial inflammatory response would be of great public health benefit.

The postprandial inflammatory response is influenced by the fat content and composition of the test meal^(^^10^^–^^13^^)^. It has been shown that SFA induce inflammation postprandially^(^^8^^)^. Palmitic acid in particular appears to be detrimental. The consumption of palmitic acid acutely increases the insulin response compared with oleic acid and *n*-3 fatty acids^(^^14^^)^ and this was shown to be related to the adverse effects of palmitate on both β-cell function and insulin sensitivity in the postprandial state^(^^15^^)^. Palmitate, either as an isolated fatty acid or as part of a high-SFA meal, increases inflammatory markers in the postprandial state^(^^16^^)^. Palm oil (PO) is enriched in palmitate and has been used widely in the food industry as a substitute for *trans*-fatty acids, which are known to have deleterious effects on CVD risk^(^^17^^)^.

Milk fat globule membrane (MFGM) fractions have previously been reported to reduce inflammation in *in vitro* and animal models^(^^18^^–^^21^^)^. MFGM, a protein–lipid complex originating from the apical surface of mammary epithelial cells, surrounds the fat globules in milk and is found in dairy products at varying levels. Specific proteins and lipids of MFGM are associated with health-promoting bioactive functions. For example, one major MFGM-associated protein, lactadherin, was reported to bind and neutralise viruses, reduce intestinal inflammation, improve intestinal permeability, and repair intestinal epithelium^(^^20^^,^^22^^–^^24^^)^. MFGM-derived polar lipids were reported to have bactericidal properties, bind enterotoxigenic pathogens and reduce intestinal inflammation^(^^19^^,^^23^^,^^25^^–^^27^^)^. In addition to functions attributed to its individual components, MFGM as a complex also reduced inflammation *in vitro*, in animals and clinically^(^^18^^,^^21^^,^^28^^)^. However, it is not known whether the addition of a dairy fraction rich in MFGM to meals high in saturated fat would blunt the postprandial inflammatory response in human subjects.

The objective of this study was to determine the postprandial inflammatory effect of a high-saturated fat meal using PO with and without the addition of MFGM in overweight and obese adults. We hypothesised that consuming a high-fat PO + MFGM meal would result in lower pro-inflammatory serum markers compared with the isoenergetic PO meal.

## Materials and methods

### Participants

A total of seventeen adult men and nineteen adult women (total of thirty-six participants) were recruited from the Davis and greater Sacramento areas of California to participate in this study. To qualify, individuals had to be between 18 and 65 years of age, and either be overweight according to their BMI (25–29·9 kg/m^2^) plus have two or more MetS traits according to the definition of the American Heart Association or simply be obese according to their BMI (30–39·9 kg/m^2^) and have any number of MetS traits. The MetS is defined by having three or more of the following traits: waist circumference >40 inches (>102 cm) for men and 35 inches (>89 cm) inches for women; fasting plasma TAG ≥ 150 mg/dl (≥ 1·70 mmol/l); fasting plasma HDL-cholesterol <40 mg/dl (<1·04 mmol/l) for men and <50 mg/dl (<1·30 mmol/l) for women; blood pressure ≥130/85 mmHg; and fasting glucose ≥100 mg/dl (≥5·56 mmol/l)^(^^29^^)^. Individuals were excluded from participation for the following reasons: diagnosis of immune-related diseases, gastrointestinal disorders, cancer, type 2 diabetes, eating disorder, allergies to the provided study foods, poor vein accessibility according to the research phlebotomist, or a body-weight change greater than 10 % over the past 6 months. Individuals were excluded from participation if they used the following: weight loss medications; daily non-steroidal anti-inflammatory drugs (NSAID); anti-inflammatory supplements; corticoid steroids; tobacco; change in hormonal birth control regimen with the past 6 months; initiation of statins in past 3 months. Because of possible confounding effects on inflammatory outcomes, dietary exclusion criteria were as follows: >1 serving of fish/week; >14 g fibre/1000 kcal (4184 kJ) per d; <16:1 of total dietary *n*-6:*n*-3 ratio; >1 % of daily energy as *trans*-fats; and a vegetarian diet pattern. If individuals had initiated an exercise programme within the past 6 months, planned to become pregnant within the next 6 months, or were already pregnant or lactating, they were not enrolled in the study. To determine enrolment eligibility, questionnaires were administered regarding health history, diet and medication. An online FFQ was used to assess dietary intake and a fasting blood sample was drawn for the analysis of blood lipids and glucose. Additional anthropometric measurements were taken during the screening visit to determine MetS traits (weight, height, and waist circumference).

This study was approved from an ethical standpoint by the Institutional Review Board of the University of California, Davis. Informed consent was given in writing by all study participants prior to starting the study protocol. The study was registered at clinicaltrials.gov under NCT01811329.

### Study design

Two isoenergetic test meals were consumed by the participants in a randomised, double-blinded, two-way cross-over design. A high-fat PO meal was compared against a high-fat PO meal with the addition of MFGM (PO + MFGM). Participants were assigned to test meal order using a random number generator which randomly returned either a 0 or 1, with a 0 being assigned to PO first followed by PO + MFGM second, and a 1 being assigned to PO + MFGM first followed by PO second. Test meals were consumed in random order on different test days separated by a washout phase of minimally 1 week and maximally 2 weeks to avoid any carry-over effects. After each washout, participants consumed the alternate test meal.

To limit confounding effects, the consumption of anti-inflammatory supplements, alcohol or NSAID was not permitted for 72 h before each test day. At 24 h prior to the test day, vigorous exercise was prohibited to avoid increasing inflammatory markers and conversely consumption of seafood was not allowed to avoid a suppression of inflammatory markers. To ensure compliance, participants filled out a 1-d food record for the 24 h prior to each test day. The dietary records were analysed using the Nutrition Data System for Research (NDSR; University of Minnesota).

Participants arrived at the Western Human Nutrition Research Center after a 10–12 h fast on each test day. The 24 h diet record was collected and participants were asked to complete a modified gastrointestinal questionnaire^(^^30^^)^. A fasted blood sample was drawn via venepuncture. Blood pressure, heart rate, weight and waist circumference were measured. The dietary test meal was then consumed completely within 20 min. Postprandial blood draws were conducted at 1, 3 and 6 h. These time points were determined based on previous postprandial clinical trials observing a peak in pro-inflammatory cytokine concentrations around 3–6 h after consuming a high-fat meal^(^^6^^,^^31^^)^.

Consumption of any food other than the test meal was not permitted, but bottled water was offered throughout the test day. Participants were offered the option to stay at the research facility or leave between blood draws via car to limit physical activity and had to return 15 min before their scheduled blood draw to allow for a 10 min rest period prior to each blood draw.

### Dietary challenges

The two test meals were made up of a bagel with strawberry preserves along with either a PO or PO + MFGM smoothie. In each instance the smoothie consisted of deionised water, cream of tartar, PO shortening, and raspberry sorbet. Additionally, the PO + MFGM smoothie contained BPC50, a cream-derived complex milk lipid fraction powder (β serum concentrate) that is a proprietary product supplied by Fonterra Co-operative Group Ltd (New Zealand)^(^^32^^)^. BPC50 is comprised of the following (% w/w): 52 % protein of which 13·2 % is membrane-derived protein, 6·6 % lactose and 36·2 % total fat (22·5 % TAG and 13·7 % phospholipids), 0·63 % gangliosides (GD3), and 5·2 % ash^(^^33^^–^^35^^)^. The six highest abundant MFGM-derived proteins reported in BPC50 include: fatty acid-binding protein, butyrophilin, lactadherin, adipophilin, xanthine oxidase and mucin^(^^35^^)^. The PO smoothie contained whey protein isolate to match the protein content found in the BPC50 product. For ingredient details, see Supplementary Table S1. Participants were instructed to eat the entire meal, rinse their cup with water, and drink the rinse-water.

Each test meal provided 40 % of the participant's total daily energy intake. Energy intake was determined by using the National Academy of Sciences equation from the Institute of Medicine Dietary Reference Intake^(^^36^^)^. To determine each participant's physical activity level the Baecke Physical Activity questionnaire was used^(^^37^^)^.

The two isoenergetic test meals were constructed to vary less than 0·2 % in macronutrients and provided about 55 % fat, about 30 % carbohydrates, and about 15 % protein. Each test meal provided between 49 and 87 g of fat depending on each individual's energy intake, 61–107 g of carbohydrates, and 31–55 g of protein (Table 1). Test meal nutrient composition was estimated using NDSR (University of Minnesota). The addition of MFGM (ranging from 53·2 to 93·1 g depending on each individual's energy intake) replaced 31 % of the fat of each participant's meal (34 % of the total energy).

**Table 1.** Nutrient composition of test meals† (Mean values and standard deviations)

	PO meal		PO + MFGM meal	
	Mean	sd	Mean	sd
Energy (kcal)	1088·1	189·3	1088·1	189·2
Energy (kJ)	4552·4	791·8	4552·7	791·7
Total carbohydrate				
g	82·7	14·4	83·2	14·5
% total energy	30·4	0·0	30·6	0·0
Total protein				
g	42·5	7·4	43	7·5
% total energy	15·6	0·0	15·8	0·0
Total fat				
g	67·3	11·7	67·5	11·7
% total energy	55·6	0·0	55·8	0·0
Total SFA				
g	32·6	5·7	34·6	6·0
% total energy	27·0	0·0	28·6	0·0
Total MUFA				
g	24·6	4·3	22·7	3·9
% total energy	20·3	0·0	18·8	0·0
Total PUFA*				
g	6·9	1·2	6·1	1·1
% total energy	5·7	0·0	5·1	0·0
SFA 4 : 0 (butyric acid) (%)	0	0	0·2	0·0
SFA 6 : 0 (caproic acid) (%)	0	0	0·2	0·0
SFA 8 : 0 (caprylic acid) (%)	0	0	0·2	0·0
SFA 10 : 0 (capric acid) (%)*	0	0	0·5	0·1
SFA 12 : 0 (lauric acid) (%)*	0·1	0·0	0·8	0·1
SFA 14 : 0 (myristic acid) (%)*	0·7	0·1	2·5	0·4
SFA 16 : 0 (palmitic acid) (%)*	28·7	5	24·8	4·3
SFA 18 : 0 (stearic acid) (%)*	2·9	0·5	4·7	0·8
MUFA 16 : 1 (palmitoleic acid) (%)*	0·2	0·0	0·5	0·1
MUFA 18 : 1 (oleic acid) (%)*	24·3	4·2	22·2	3·9
PUFA 18 : 2 (linoleic acid) (%)	6·6	1·1	5·1	0·9
PUFA 18 : 3 (linolenic acid) (%)	0·3	0·1	0·5	0·1

PO, palm oil; PO + MFGM, palm oil + milk fat globule membrane.

* Significant difference between the two meals (*P* < 0·05).

† Comparison of the dietary challenges. Nutrient composition obtained using the Nutrition Data System for Research (NDSR). Test meals were based on each individual's total energy expenditure; thus values shown are average of all test meals (*n* 36).

### Blood analyses

Whole blood was drawn at baseline, and at 1, 3 and 6 h after the meal. Serum tubes were allowed to clot at room temperature for 30 min, and then centrifuged at 1300 ***g*** at 4°C for 10 min. EDTA-whole blood tubes were kept on ice during and after blood collection and were centrifuged within 30 min of collection at 1300 ***g*** at 4°C for 10 min. After centrifugation, the serum and plasma tubes were kept on ice during aliquoting. Subsequently, plasma and serum aliquots were directly frozen at –70°C until analysed.

### Inflammatory markers

Serum samples from all four time points were analysed for cytokine concentrations (IL-10, IL-1β, IL-2, IL-4, IL-6, IL-8, TNFα, monocyte chemotactic protein-1), as well as the vascular injury molecules C-reactive protein (CRP), serum amyloid A, soluble intracellular adhesion molecule (sICAM) and soluble vascular adhesion molecule. Plasma was used to measure IL-18 concentrations. A commercially available Multi Spot ELISA kit was used to quantify the concentrations of these markers (SECTOR Imager 2400; Meso Scale Discovery). The protocol was followed as recommended by the manufacturer. Briefly, pre-coated plates with capture antibodies were incubated with 25–50 µl of serum or plasma. After washing the plates a labelled detection antibody was added. Upon electrochemical stimulation the bound detection antibodies emit light, which is measured by the plate reader to quantify the amount of each protein of interest.

### Cortisol

Serum cortisol was measured at all times points using the DetectX Cortisol Enzyme Immunoassay kit (Arbor Assays). Briefly, a cortisol–peroxidase conjugate and a monoclonal cortisol antibody were added to a pre-coated ninety-six-well plate. Upon incubation, serum samples were added to each well and allowed to bind with the cortisol–peroxidase conjugate. The total amount of cortisol present in each sample was then calculated based on the absorbance detected by the reader.

### Metabolic parameters

At each time point plasma glucose, insulin, and a lipid panel including TAG, total cholesterol, HDL-cholesterol, LDL-cholesterol, HDL:LDL ratio, and non-HDL-cholesterol were assessed by standard clinical techniques in the clinical laboratory of University of California Medical Center (Sacramento, CA).

### Clinical characteristics

Body weight, height, waist circumference, blood pressure and heart rate were measured on each test day. Body weight was measured with a calibrated scale (6002 Wheelchair Scale; Scale-tronix). Waist circumference was measured in the standing position with measurements midway between the lateral lower rib margin and the ileac crest (QM2000 Measure Mate; QuickMedical). Height was measured with a wall-mounted stadiometer (Ayrton Stadiometer Model S100; Ayrton Corporation). Blood pressure and resting heart rate were taken in the upright seated position using the appropriately sized cuff (Carescape V100 with Critikon Dura-cuf for either adults or large adults; GE Medical Instruments). Total fat mass and lean mass were assessed using dual-energy X-ray absorptiometry (Lunar Prodigy instrument; GE Medical Instruments).

### Statistical analysis

The sample size was calculated based on the primary outcome marker IL-6 using the means and standard deviations from a similar human study with overweight men at risk for developing the MetS^(^^31^^)^. To ensure 95 % confidence of the results and 80 % power the sample size calculation indicated that thirty-six participants would be needed.

Statistical analyses were conducted on SPSS version 20.0 software for Macintosh (SPSS). Differences were considered significant at *P* < 0·05. Normality was established visually and numerically using histograms, Q–Q plots and the Shapiro–Wilk test. Data were transformed as needed. When concentrations for markers were below the lower limit of detection (LLOD) (IL-10; 23 % and IL-6; 9 %) for <25 % of the samples, the value was calculated as the LLOD divided by 10. When concentrations for markers were below the LLOD for >25 % of the samples, the data were excluded from statistical analyses (IL-1β and IL-4). Cases with values more than three box lengths from the 75th percentile or 25th percentile were deemed outliers and removed from all analyses; this only applied to sICAM where two subjects were excluded.

To determine if dietary differences existed between test meal composition and baseline analyte concentrations a paired *t* test was used. A mixed linear model was performed with treatment and time as fixed factors, participants as the random effect and treatment × time as the interaction term. If time was significant, multiple-comparison *post hoc* analysis with Bonferroni correction was carried out to compare the concentrations at 0–1 h, 0–3 h, 0–6 h, 1–3 h, 1–6 h, and 3–6 h.

The incremental AUC (iAUC, area above baseline) and decremental (area below baseline) using the conventional trapezoid method were used to compare postprandial responses between test meals^(^^38^^)^. The iAUC was chosen over the total AUC because it reflects the postprandial rise of these metabolite concentrations above the non-zero fasting value^(^^39^^)^. iAUC between test meals were compared by one-way ANOVA.

To determine if pre-existing clinical conditions affected the inflammatory responses to the test meals, secondary analyses were conducted. Participants were coded as having high or low CRP levels based on their baseline levels prior to receiving either test meal treatment. High CRP was defined as a concentration ≥3 mg/l (*n* 17), low CRP was defined as <3 mg/l (*n* 19)^(^^40^^)^. Baseline characteristics of each CRP group can be found in Table 2. ANCOVA was used to identify statistically significant differences in postprandial inflammatory markers between test meals using CRP as the covariate variable.

**Table 2.** Participant baseline characteristics* (Mean values and standard deviations)

	All participants			Low CRP‡		High CRP‡	
	Mean	sd	MetS criteria†	Mean	sd	Mean	sd
Age (years)	42·9	14·0		42·4	13·6	43·6	14·9
Weight (kg)	92·9	12·2		93·5	11·9	92·2	12·8
Height (m)	1·7	0·1		1·7	0·1	1·7	0·1
BMI (kg/m^2^)	31·7	2·6		31·9	2·6	31·5	2·5
Total body fat (%)	36·7	7·8		34·2	8·3	39·5	6·3
Total body fat, male (%)§	31·0	6·2		28·5	4·3	35·5	6·8
Total body fat, female (%)§	41·9	4·9		42·1	5·1	41·7	5·0
Android fat (g)	3262·9	799·9		40·5	6·7	46·1	4·2
Android fat, male (g)	3370·8	934·1		37·2	5·7	45·2	3·3
Android fat, female (g)	3166·3	669·0		45·0	5·3	46·7	4·7
Gynoid fat (g)	5353·3	1452·0		36·9	8·6	39·5	7·0
Gynoid fat, male (g)	4829·2	1325·8		31·2	5·0	33·5	5·3
Gynoid fat, female (g)	5822·1	1430·2		44·8	5·7	42·7	5·6
WC (inches)	39·3	3·2		38·8	2·9	39·9	3·4
WC, male (inches)	41·1	3·1	>40	40·2	2·9	42·8	2·9
WC, female (inches)	37·7	2·2	>35	36·9	1·6	38·2	2·4
WC (cm)	99·8	8·1		98·6	7·4	101·3	8·6
WC, male (cm)	104·4	7·9	>102	102·1	7·4	108·7	7·4
WC, female (cm)	95·8	5·6	>89	93·7	4·1	97·0	6·1
Systolic BP (mmHg)	123·9	13·6	≥130	123·1	9·5	124·8	17·4
Diastolic BP (mmHg)	75·0	10·3	≥85	76·0	9·2	73·9	11·6
HDL-cholesterol							
mg/dl	48·3	14·1		48·4	14·2	48·2	14·4
mmol/l	1·25	0·37		1·25	0·37	1·25	0·37
HDL-cholesterol, male							
mg/dl	43·2	11·7	<40	42·5	13·7	44·5	7·8
mmol/l	1·12	0·30	<1·04	1·10	0·35	1·15	0·20
HDL-cholesterol, female							
mg/dl	52·9	14·7	<50	56·5	10·9	50·3	16·9
mmol/l	1·37	0·38	<1·30	1·46	0·28	1·30	0·44
Fasting glucose							
mg/dl	91·0	7·4	≥100	92·0	7·8	89·9	7·1
mmol/l	5·06	0·41	≥5·56	5·11	0·43	4·99	0·39
Fasting TAG							
mg/dl	122·5	57·8	≥150	106·0	41·1	140·9	68·7
mmol/l	1·38	0·65	≥1·70	1·20	0·46	1·59	0·78

MetS, metabolic syndrome; CRP, C-reactive protein; WC, waist circumference; BP, blood pressure.

* Measurements taken at screening visit (*n* 36).

† MetS as defined by the American Heart Association.

‡ Low baseline CRP *n* 19; high baseline CRP *n* 17.

§ Male *n* 17, female *n* 19.

## Results

### Participant characteristics

**Fig. 1.**
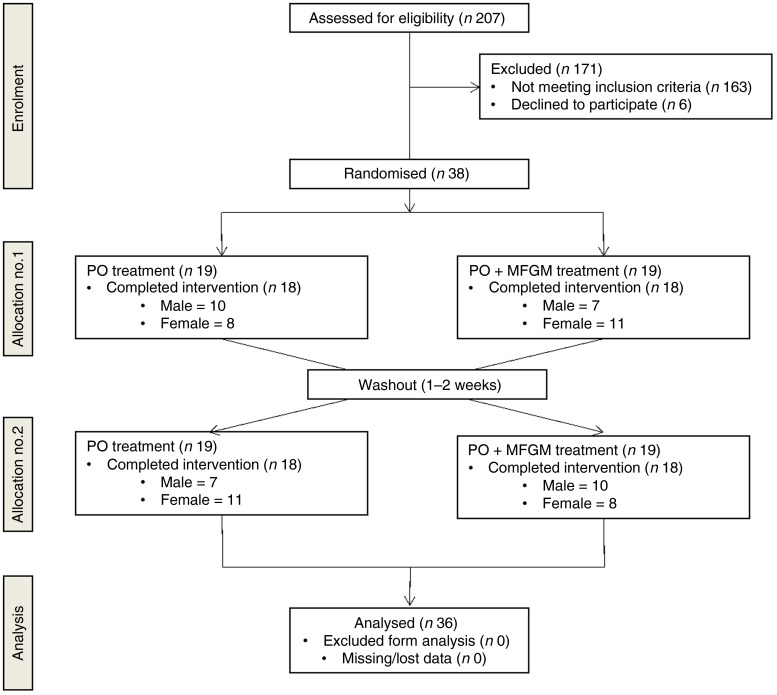
Enrolment and follow-up of participants in the randomised cross-over trial. PO, palm oil; PO + MFGM, palm oil + milk fat globule membrane.

After screening 207 participants, thirty-eight were enrolled to start the study (Fig. 1). Thirty-six participants completed both postprandial test days. The two participants who did not finish the trial were disqualified due to scheduling difficulties and the initiation of medication that could confound the results. Each participant was randomly assigned to one test meal and after a 1- to 2-week washout period, they were crossed-over to the alternate test meal. The majority of the study population was Caucasian (67 %) or Hispanic (28 %). Out of the total thirty-six participants, six were overweight with two MetS traits, three were overweight with three or more MetS traits, twenty-one were obese with zero to two MetS traits, and six were obese with three MetS traits. The baseline characteristics of the participants are shown in Table 2.

### Dietary challenge

Participants consumed two test meals, a high-fat PO test meal and a high-fat PO + MFGM test meal. The meals (Table 1) were isoenergetic and comparable for macronutrient composition, not varying by more than 0·2% for carbohydrates, protein or fat. The total weight of SFA and MUFA did not differ between the PO *v*. PO + MFGM test meals. However, the PO meal contained a significantly higher total amount of PUFA compared with the PO + MFGM test meal. The relative abundance of 18 : 2*n*-6 was not significantly different between the two test meals; however, since 18 : 2*n*-6 is the predominant PUFA in the two meals, and since the PO meal had higher total PUFA, the PO meal had a higher total amount of 18 : 2*n*-6. The relative abundances of specific SFA and MUFA were significantly different: the PO + MFGM meal had more 10 : 0, 12 : 0, 14 : 0, 18 : 0 and 18 : 1*n*-9 whereas the PO meal had more 16 : 0 and 16 : 1*n*-7. These differences in relative abundances of fatty acids are reflective of the composition of PO, which is enriched in palmitate (16 : 0), and MFGM, which is enriched in medium-chain SFA characteristic of dairy fat.

### Metabolic parameters

There was a time × treatment interaction for total cholesterol (*P* = 0·04), HDL-cholesterol (*P* = 0·01), TAG (*P* < 0·0005), non-HDL-cholesterol (*P* = 0·04) and insulin (*P* < 0·0005) (Table 3). Among these lipid makers, the greater change in total cholesterol was observed in response to the PO test meal; there was a 5 % increase from 0 to 1 h as well as from 0 to 6 h. HDL-cholesterol increased from 0 to 1 h by 4 % and decreased from 1 to 3 h by 4 % in response to the PO test meal. In response to the PO + MFGM test meal, TAG concentration increased by 104 % from 0 to 3 h and non-HDL-cholesterol concentration increased by 22 % from 0 to 1 h.

**Table 3.** Concentrations of metabolic markers with significant interaction effects (Mean values and standard deviations)

	Time point								
	0 h		1 h		3 h		6 h		
	Mean	sd	Mean	sd	Mean	sd	Mean	sd	Time × treatment: *P*
Total cholesterol (mg/dl)									0·04
PO meal	194·9	38·5	204·2*	40·8	201·4*	39·9	203·9*	39·6	
PO + MFGM meal	198·7	37·6	199·7	37·6	207·1	37·4	203·7	35·8	
Total cholesterol (mmol/l)									0·04
PO meal	5·05	1·00	5·29*	1·06	5·22*	1·03	5·28*	1·03	
PO + MFGM meal	5·15	0·97	5·17	0·97	5·36	0·97	5·28	0·93	
HDL-cholesterol (mg/dl)									0·01
PO meal	49·6	14·5	51·6*	15·3	49·3†	15·3	49·9†	15·3	
PO + MFGM meal	50·2	14·0	50·6	14·5	50·4	14·5	49·7	14·4	
HDL-cholesterol (mmol/l)									0·01
PO meal	1·28	0·37	1·34*	0·40	1·28†	0·40	1·29†	0·40	
PO + MFGM meal	1·30	0·36	1·31	0·38	1·30	0·37	1·29	0·37	
TAG (mg/dl)									<0·0005
PO meal	126·6	56·5	182·9*	77·9	223·9*†	114·4	209·6*‡	82·9	
PO + MFGM meal	130·1	70·6	174·9	58·2	265·1	119·8	210·4	111·8	
TAG (mmol/l)									<0·0005
PO meal	1·43	0·64	2·07*	0·88	2·53*†	1·29	2·37*‡	0·94	
PO + MFGM meal	1·47	0·80	1·98	0·66	3·00	1·35	2·38	1·26	
Non-HDL-cholesterol (mg/dl)									0·04
PO meal	145·4	35·3	152·6*	36·5	152·2*	36·3	154·0*	37·2	
PO + MFGM meal	148·5	34·9	180·5	166·6	157·0	34·5	154·0	33·8	
Non-HDL-cholesterol (mmol/l)									0·04
PO meal	3·77	0·91	3·95*	0·95	3·94*	0·94	3·99*	0·96	
PO + MFGM meal	3·85	0·90	4·67	4·32	4·07	0·89	3·99	0·88	
Insulin (μIU/ml)									<0·0005
PO meal	14·0	6·1	81·7*	69·3	34·4*†	28·6	Not measured		
PO + MFGM meal	16·4	10·6	54·8	46·1	39·6	25·9	Not measured		
Insulin (pmol/l)									<0·0005
PO meal	100·7	44·1	586·4*	497·4	246·5*†	205·5	Not measured		
PO + MFGM meal	117·5	75·9	392·9	330·4	283·8	186·0	Not measured		

PO, palm oil; PO + MFGM, palm oil + milk fat globule membrane.

* Significantly different from 0 h when both treatments analysed together (*P* < 0·05).

† Significantly different from 1 h when both treatments analysed together (*P* < 0·05).

‡ Significantly different from 3 h when both treatments analysed together (*P* < 0·05).

The total concentration of each analyte over the 6 h postprandial time was calculated as the iAUC and compared between test meals. The addition of MFGM to the test meal resulted in significantly lower concentrations of total cholesterol (*P* = 0·02 with all subjects included, *P* = 0·04 with two outliers removed), LDL-cholesterol (*P* = 0·046) and a significantly higher concentration of TAG (*P* = 0·025) when compared with the PO meal alone (Supplementary Fig. S1).

**Fig. 2.**
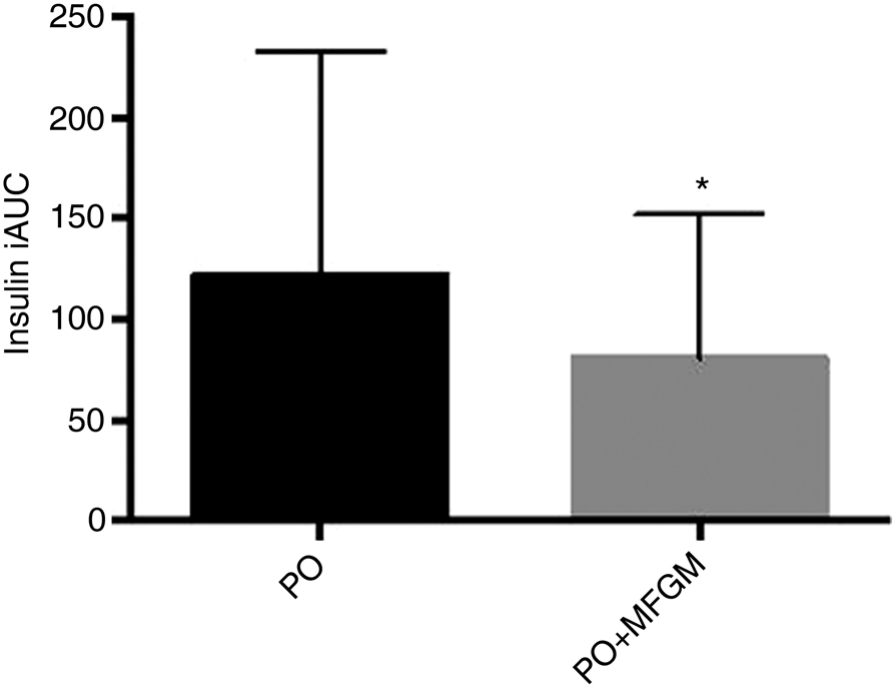
Postprandial serum insulin concentrations. Serum insulin concentrations over the 6 h postprandial period after a high-fat mixed meal containing palm oil (PO) *v*. palm oil + milk fat globule membrane (PO + MFGM). Data are incremental AUC (iAUC). Values are means, with standard deviations represented by vertical bars. * The addition of MFGM resulted in a significant decrease of insulin concentration (*P* = 0·005).

When total insulin concentration was compared over the 6 h postprandial period (i.e. the iAUC of insulin from 0 to 6 h) the addition of MFGM resulted in a significantly lower exposure to insulin (*P* = 0·005) (Fig. 2). Neither insulin nor glucose concentrations at baseline differed between the two test meals. There were no effects of treatment on insulin and glucose concentrations in the postprandial period; however, there was a rapid increase in insulin concentration from 0 to 1 h (*P* < 0·0005) that was complemented by a decrease in glucose (*P* < 0·0005). From 1 to 3 h there was an increase back to baseline levels for glucose (*P* = 0·003) and a corresponding decrease in insulin concentrations (*P* < 0·0005) although still greater than the 0 h value (*P* < 0·0005). The increase in insulin concentration was dampened from 0 to 1 h by more than 50 % when MFGM was added to the test meal, resulting in a 482 % increase *v*. a 234 % increase for PO *v*. PO + MFGM, respectively (time × treatment effect, *P* < 0·0005).

**Fig. 3.**
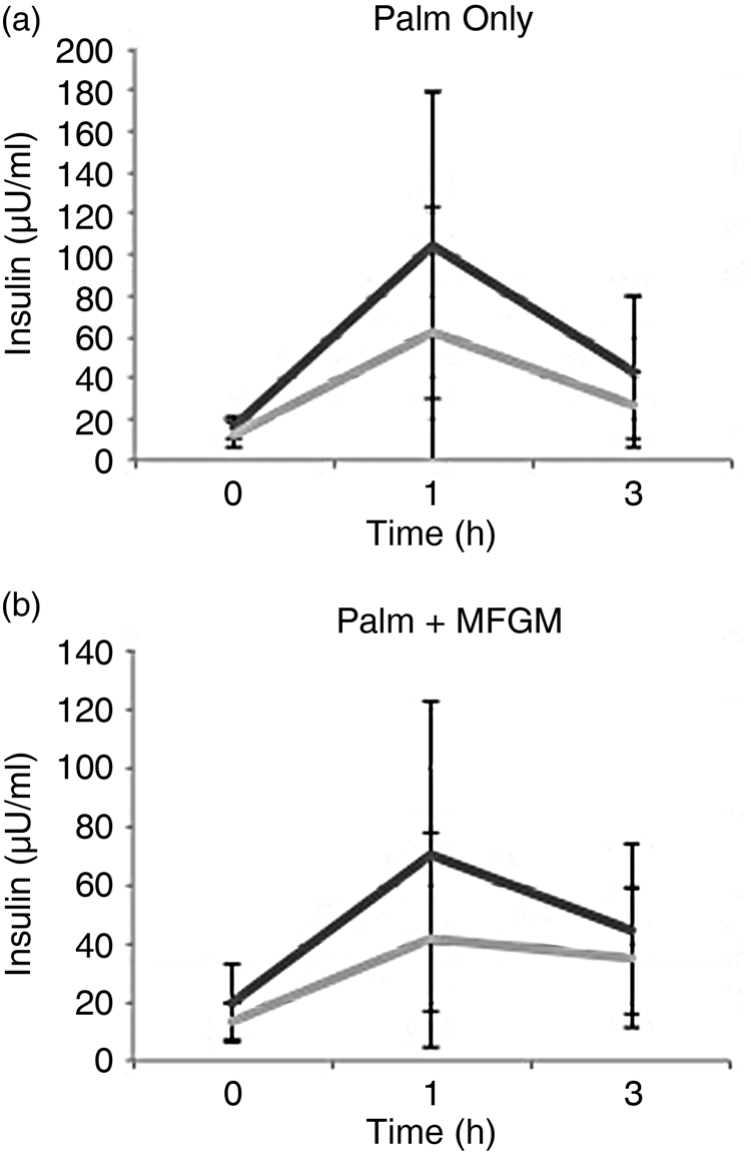
Insulin concentrations in high (––) *v*. low (––) baseline C-reactive protein (CRP) groups. (a) There was a significant difference between the high and low baseline CRP groups at the 1 h time point (*P* = 0·03) after consuming the palm oil meal. (b) When the palm oil + milk fat globule membrane meal was consumed there was no difference between the high and low baseline CRP groups for insulin. Values are means, with standard deviations represented by vertical bars. To convert insulin to pmol/l, multiply by 6·945.

To examine if there was a difference between participants who had high *v*. low baseline CRP concentrations, additional secondary analyses were conducted. Participants with baseline CRP concentration ≥3 mg/l (coded as ‘high’) had significantly higher insulin concentrations at 1 h after consuming the PO meal (*P* = 0·03). The addition of MFGM to the meal suppressed the insulin response in the high CRP group, thus removing any significant difference between the high and low CRP groups (Fig. 3).

### Inflammatory markers

The two cytokines IL-1β and IL-4 fell below the detection limit for 70 and 95 % of samples, respectively. Consequently, these markers were not included in the statistical analyses reported here. Similar results for IL-1β and IL-4 have been observed in previous studies^(^^41^^,^^42^^)^.

**Fig. 4.**
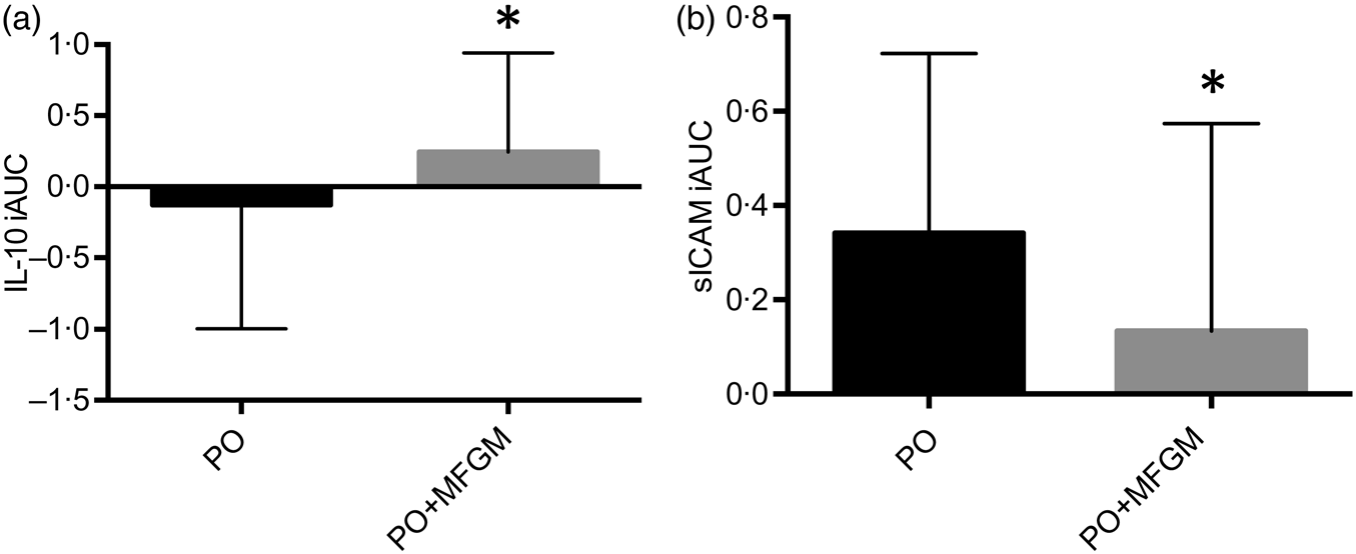
Postprandial serum concentrations of IL-10 (a) and soluble intracellular adhesion molecule (sICAM) (b). Serum IL-10 and sICAM concentrations over the 6 h postprandial period after a high-fat mixed meal containing palm oil (PO) *v*. palm oil + milk fat globule membrane (PO + MFGM). Data are incremental AUC (iAUC). Values are means, with standard deviations represented by vertical bars. (a) * The addition of MFGM resulted in a significant increase of anti-inflammatory IL-10 (*P* = 0·011). (b) * The addition of MFGM resulted in a significant decrease of sICAM concentration (*P* = 0·013). The sICAM graph and data exclude two subjects who were deemed outliers with values more than three box lengths away from the 75th or 25th percentile.

Baseline concentrations of all markers related to inflammation were comparable between the two treatments. When analysed as iAUC, IL-10 was significantly higher (*P* = 0·013) and sICAM was significantly lower in response to the PO + MFGM test meal (*P* = 0·005 for all) (Fig. 4). An interaction effect between time and treatment was observed for IL-10 (*P* = 0·03), IL-8 (*P* = 0·04) and sICAM (*P* = 0·02) (Table 4). Over time IL-10 gradually, but not significantly, declined on the PO treatment whereas on the PO + MFGM treatment IL-10 increased. There was a significant decrease in IL-8 from 0–3 h and 1–3 h and a significant increase in concentration from the 3–6 h time points (*P* < 0·05 for all) following the PO meal, but IL-8 was unchanged after the PO + MFGM meal. Concentrations for sICAM significantly increased from 0–1 h, 0–6 h, and 3–6 h and significantly decreased between the 1–3 h time points (*P* < 0·05 for all) after consumption of the PO meal and, like IL-8, was unchanged after the PO + MFGM challenge. There was no treatment effect observed for any of the other inflammatory markers, but a significant change over time was observed for IL-6, IL-8, TNFα, CRP, serum amyloid A, sICAM, soluble vascular adhesion molecule and cortisol (*P* < 0·05 for all) (Table 4). There was no time or treatment effect for monocyte chemotactic protein-1.

**Table 4.** Concentrations of measured inflammatory markers at each time point (Mean values and standard deviations)

	Time point								
	0 h		1 h		3 h		6 h		
	Mean	sd	Mean	sd	Mean	sd	Mean	sd	Time × treatment: *P*
IL-10 (pg/ml)									
PO meal	0·56	1·35	0·54	1·34	0·54	1·20	0·51	1·30	0·03
PO + MFGM meal	0·49	1·15	0·52	1·16	0·52	1·09	0·57	1·10	
IL-6 (pg/ml)									
PO meal	0·74	1·07	0·59*	0·86	0·61*	1·08	0·76†‡	1·14	0·48
PO + MFGM meal	0·72	1·35	0·59	1·23	0·54	0·95	0·76	1·36	
IL-8 (pg/ml)									
PO	11·24	3·06	10·51*	3·30	9·63*	3·11	10·96	2·91	0·04
PO + MFGM	11·11	3·28	11·32	3·23	10·72	2·66	10·8	3·30	
TNFα (pg/ml)									
PO meal	2·49	0·70	2·36	0·56	2·29	0·62	2·36	0·64	0·13
PO + MFGM meal	2·34	0·66	2·36	0·64	2·27	0·61	2·22	0·66	
IL-18 (pg/ml)									
PO meal	145·48	91·64	148·44	75·95	162·38	109·73	150·95	73·05	0·59
PO + MFGM meal	154·25	83·16	152·19	73·41	155·38	78·06	160·03	72·09	
MCP-1 (pg/ml)									
PO meal	345·75	93·66	343·23	104·24	336·72	94·82	330·81	82·09	0·26
PO + MFGM meal	352·16	84·36	358·61	90·41	320·46	102·57	335·05	97·92	
CRP (mg/l)									
PO meal	4·42	4·69	4·39	4·23	4·42	4·43	4·64†	4·64	0·8
PO + MFGM meal	4·37	4·29	5·16	5·56	5·11	5·54	5·20	5·38	
SAA (mg/l)									
PO meal	3·93	3·48	4·04	3·58	3·81†	3·22	4·12‡	3·71	0·91
PO + MFGM meal	11·85	30·79	12·76	32·76	12·20	31·68	12·82	33·37	
sICAM-1 (mg/l)									
PO meal	0·97	0·50	1·06*	0·53	1·00†	0·50	1·04*‡	0·53	0·02
PO + MFGM meal	0·96	0·51	0·97	0·50	0·95	0·50	0·97	0·51	
sVCAM-1 (mg/l)									
PO meal	1·55	0·85	1·65*	0·86	1·58†	0·86	1·62‡	0·86	0·13
PO + MFGM meal	1·49	0·83	1·51	0·84	1·46	0·82	1·52	0·85	
Cortisol (μg/l)									
PO meal	1139751	820859	967108	659595	703305*†	457081	653937*†‡	577352	0·76
PO + MFGM meal	1083979	698219	933085	619060	780686	476281	613297	366507	

PO, palm oil; PO + MFGM, palm oil + milk fat globule membrane; MCP-1, monocyte chemoattractant protein-1; CRP, C-reactive protein; SAA, serum amyloid A; sICAM, soluble intracellular adhesion molecule; sVCAM, soluble vascular adhesion molecule.

* Significantly different from 0 h when both treatments analysed together (*P* < 0·05).

† Significantly different from 1 h when both treatments analysed together (*P* < 0·05).

‡ Significantly different from 3 h when both treatments analysed together (*P* < 0·05).

Secondary analysis using baseline CRP as a covariate revealed a statistically significant difference for IL-6 at each time point (*P* < 0·05 for each time point) between participants with high *v*. low baseline CRP concentrations after consuming the PO test meal. After consuming the PO + MFGM test meal, this difference was no longer significant, suggesting that MFGM may attenuate the postprandial inflammatory response in individuals with high CRP levels.

### Cortisol

Analysis of serum cortisol revealed no time × treatment interaction effect or a treatment effect. There was a significant decrease in cortisol concentration for all time points (*P* < 0·05 for all time points) over the course of each test day. Decreases throughout the day, from baseline to 1 h, 1–3 h, and 3–6 h were as follows: 15, 33 and 43 %, respectively. This observed decrease throughout the day is consistent with diurnal patterns of cortisol.

## Discussion

This study was designed to determine if the addition of MFGM to a high-fat meal containing plant-based saturated fat influences postprandial inflammation in overweight and obese individuals. Our results showed that adding a dairy fraction rich in MFGM to a high-fat meal may lower CVD risk by reducing postprandial insulin, total cholesterol and LDL-cholesterol as well as sICAM concentrations while increasing the concentration of anti-inflammatory IL-10.

Compared with PO, consumption of the PO + MFGM test meal resulted in a significantly higher concentration of IL-10 at 6 h postprandial. IL-10 is an anti-inflammatory cytokine which has been recognised for its atheroprotective effects^(^^43^^)^. Our results suggest that the addition of MFGM to a high-SFA meal improves postprandial inflammation in an overweight and obese population already in a chronically inflamed state. To our knowledge we are the first to examine the postprandial effect on IL-10 after a high-fat dietary challenge with and without MFGM in human subjects.

Cellular adhesion molecules, such as sICAM, are key players in the early events of atherosclerosis development^(^^44^^)^. The consumption of the PO + MFGM test meal resulted in a significantly lower concentration of sICAM over the postprandial period when compared with PO alone. In large prospective studies of both healthy individuals and patients with CVD, concentrations of sICAM were positively associated with future incidents of CVD^(^^45^^–^^47^^)^. In our study the total amount of sICAM over the 6 h postprandial period in response to PO + MFGM was significantly lower by 95 % compared with PO. These results suggest that the addition of a dairy fraction rich in MFGM attenuates the atherogenic milieu triggered by the PO meal.

It is possible that the difference in sICAM between the PO *v*. PO + MFGM treatments could stem from the difference in the fatty acid composition. The PO + MFGM meal was higher in short- and medium-chained SFA and lower in 18 : 2*n*-6 and 18 : 1*n*-9 compared with the PO meal. Chen *et al*.^(^^48^^)^ showed that when human retinal vascular endothelial cells were treated with linoleic acid (18 : 2*n*-6) it resulted in increased ICAM expression. The MFGM preparation used is composed of complex lipids including sphingolipids, as well as bioactive proteins, which may also play roles in the observed anti-inflammatory effects^(^^49^^)^.

When analysing the insulin iAUC over the 6 h postprandial period, the total insulin concentration was significantly lower in response to the PO + MFGM compared with the PO test meal. To our knowledge there have not been any prior clinical trials examining the effect of MFGM consumption on the insulin response in human subjects. One study investigated the postprandial effect of adding a dairy product rich in sphingolipids, a lipid constituent of MFGM, to a high-fat breakfast meal and found no significant difference in postprandial insulin concentrations^(^^50^^)^. Branched-chain amino acids may promote insulin secretion^(^^51^^,^^52^^)^; thus the effect of MFGM on insulin may be related to its amino acid composition.

The secondary analysis based on baseline CRP concentrations revealed that participants who were in an inflamed state (CRP ≥ 3 mg/l) in the fasted condition had significantly higher insulin concentrations after consuming the PO meal compared with those who had normal baseline CRP concentrations. However, the addition of MFGM to the high-fat test meal completely removed this difference. These results suggest an interaction between diet and phenotype, whereby consumption of MFGM by chronically inflamed individuals normalised responses to a high-fat meal to closely resemble that of a metabolically healthy profile.

Research has shown that cortisol peaks in the morning^(^^53^^)^, which was reflected in the present study, and decreases over the course of the day. Elevated levels of cortisol inhibit the synthesis of pro-inflammatory cytokines^(^^54^^)^. We hypothesised that this potential inhibition may explain the observed initial decrease in the pro-inflammatory cytokines (IL-6, IL-8, TNFα) from baseline (blood draw schedule between 08·00 and 09·00 hours) to the 3 h blood draw (scheduled between 11·00 and 12·00 hours). However, none of the correlations was significant.

In summary, the addition of a dairy fraction rich in MFGM reduced the iAUC in postprandial insulin, total cholesterol, LDL-cholesterol and sICAM responses over the 6 h postprandial period, and increased the production of the anti-inflammatory cytokine IL-10. The addition of a dairy fraction rich in MFGM also attenuated the increases in insulin at 1 h in individuals with elevated fasting CRP. Results from this study suggest that the addition of a dairy fraction rich in MFGM attenuates the negative metabolic and inflammatory effects of a high-fat meal rich in saturated fat, specifically palmitate.

## Supplementary material

For supplementary material accompanying this paper visit http://dx.doi.org/10.1017/jns.2015.42. click here to view supplementary material
