# Supplementary material for: Addition of a dairy fraction rich in milk fat globule membrane to a high-saturated fat meal reduces the postprandial insulinaemic and inflammatory response in overweight and obese adults
Source: J Nutr Sci. 2016 Mar 7;5:e14. doi: 10.1017/jns.2015.42 (PMC4791522; doi:10.1017/jns.2015.42)
Supplement: Supplementary file 1 [file S2048679015000427sup.zip › S2048679015000427sup001.doc]

**Supplementary Table S1. Test meal ingredient list**

|  | **PO^1^** | **PO+MFGM^2^** |
| --- | --- | --- |
| **Sandwich** |  |  |
| Plain Bagel Thins (Thomas, Bimbo Bakeries, Horsham, PA, USA) | ✓ | ✓ |
| Strawberry Preserves (Safeway Select, Pleasanton, CA, USA) | ✓ | ✓ |
| **Smoothie** |  |  |
| DI Water (Davis, CA, USA) | ✓ | ✓ |
| Whey Protein Isolate (BioChem, Country Life, Hauppauge, NY, USA) | ✓ |  |
| Cream of Tartar (Safeway Select, Pleasanton, CA, USA) | ✓ | ✓ |
| Palm Oil Shortening (Spectrum, Boulder, CO, USA) | ✓ | ✓ |
| BPC50 (Fontarra, Auckland, New Zealand) |  | ✓ |
| Raspberry Sorbet (Cioa Bella, Florham Park, NJ, USA) | ✓ | ✓ |
| The two test meals were isocaloric. Original to this manuscript. |  |  |
| ^1^Palm Oil |  |  |
| ^2^Palm Oil+Milk Fat Globule Membrane |  |  |
