# Supplementary material for: Addition of a dairy fraction rich in milk fat globule membrane to a high-saturated fat meal reduces the postprandial insulinaemic and inflammatory response in overweight and obese adults
Source: J Nutr Sci. 2016 Mar 7;5:e14. doi: 10.1017/jns.2015.42 (PMC4791522; doi:10.1017/jns.2015.42)
Supplement: Supplementary file 1 [file S2048679015000427sup.zip › S2048679015000427sup002.doc]

**Supplementary Fig. S1.** Concentrations over the 6 h postprandial period after a high-fat mixed meal of PO vs. PO+MFGM for A) total cholesterol, B) LDL, and C) TG. Data are mean iAUC ± SD. Total Cholesterol graph and data excludes two subjects who were deemed outliers with values more than 3 box-lengths away from the 75th or 25th percentile. iAUC, incremental area under the curve; PO, Palm Oil treatment; PO+MFGM, Palm Oil + Milk Fat Globule Membrane treatment; LDL, low density lipoprotein; TG, triglycerides.
